# Synthesis and Characterization of Emulsifiers Based on the Maillard Reaction and Its Application in Stabilized DHA Algal Oil Nanoemulsions

**DOI:** 10.3390/foods13111667

**Published:** 2024-05-26

**Authors:** Dan-Dan Yan, Bo Hu, Pan Gao, Jiao-Jiao Yin, Shu Wang, Yong Yang, Lei Tan, Chuan-Rong Hu, Dong-Ping He, Wu Zhong

**Affiliations:** 1 Key Laboratory of Edible Oil Quality and Safety for State Market Regulation, College of Food Science and Engineering, Wuhan Polytechnic University, Wuhan 430023, China; dan15530718367@163.com (D.-D.Y.); hubogeren@126.com (B.H.); gaopan925@163.com (P.G.); yinjiaojiao@whpu.edu.cn (J.-J.Y.); hcr305@163.com (C.-R.H.); hedp123456@163.com (D.-P.H.); 2 Key Laboratory for Deep Processing of Major Grain and Oil Ministry of Education, College of Food Science and Engineering, Wuhan Polytechnic University, Wuhan 430023, China; 3 Wuhan Institute for Food and Cosmetic Control, Wuhan 430012, China; wangshu8532@163.com (S.W.); yangywhzj@hotmail.com (Y.Y.); 4 Hubei Fuxing Biotechnology, Hanchuan 431608, China; bth_stone@163.com

**Keywords:** Maillard reaction, emulsification, DHA nanoemulsions, sodium caseinate, gum arabic, food stability, protein–polysaccharide complexes

## Abstract

The aim of this study was to optimize the formation of sodium caseinate (CS) and gum arabic (GA) complexes through the Maillard reaction and to evaluate their effectiveness in improving the emulsification properties and stability of docosahexaenoic acid (DHA) nanoemulsions. First, the best target polysaccharides were selected, and the best modification conditions were determined using orthogonal experiments. Secondly, the response surface experiments were used to optimize the preparation process of the emulsion. The stability, in vitro digestion characteristics, and rheological characteristics of the emulsion prepared by means of CS–GA were compared with the emulsion prepared using a whey protein isolate (WPI). After the orthogonal test, the optimal modification conditions were determined to be a reaction time of 96 h, a CS–GA mass ratio of 1:2, a reaction temperature of 60 °C, and a degree of grafting of 44.91%. Changes in the infrared (IR), Raman, ultraviolet (UV), and endogenous fluorescence spectra also indicated that the complex structure was modified. The response surface test identified the optimal preparation process as follows: an emulsifier concentration of 5 g/L, an oil-phase concentration of 5 g/L, and a homogenization frequency of five, and the emulsion showed good stability. Therefore, the use of a nanoemulsion as a nanoscale DHA algal oil delivery system is very promising for extending the shelf life and improving the stability of food.

## 1. Introduction

DHA (C_22_H_32_O_2_) is an unsaturated straight-chain fatty acid with twenty-two carbon atoms and six double bonds [1]. It has critical physiological functions in maintaining and promoting physical health and can reduce the risk of certain chronic diseases, such as cardiovascular diseases, immune response disorders, mental disorders, and inflammation [2]. Studies have found that an insufficient intake of DHA in infants can impair the development of brain cells. A severe deficiency may disrupt metabolism regulation, which can lead to intellectual disabilities [3]. DHA can only be synthesized in low quantities and, therefore, must be mostly obtained through food [4]. The sources of DHA include the following: (1) Breast milk: infants and young children obtain DHA from breast milk or milk powder [5]. (2) Nuts: some nuts are rich in α-linolenic acid, which undergoes a series of metabolic transformations in the human body to be converted into EPA and then DHA; however, the conversion efficiency is poor, typically less than 1% [6]. EPA has both anti-inflammatory and anti-thrombotic effects and helps to reduce the risk of heart disease and improve cardiovascular health. (3) Fish: some fish, mainly deep-sea fish such as sardines and tuna, acquire DHA through the bioaccumulation of phytoplankton, such as algae, in the marine food web [7]. (4) Algae: seaweed oil, primarily derived from algae and plankton, surpasses fish oil in DHA content and purity [8]. It acts as a vegan DHA source with advantages that include simple extraction, a high absorption, and good safety [9]. One study showed that the DHA content of *Chypulllegales* can reach 49.84% of its total fatty acid content [10].

As the DHA molecule contains six unsaturated double bonds, oxidation not only reduces its original nutritional value, but is also harmful to the human body [11]. Secondly, DHA is a highly hydrophobic molecule with a low water solubility [12]. DHA often loses its structural and functional integrity in acidic gastric environments, leading to a significant decrease in its bioavailability [13,14]. It is vulnerable to light and heat during processing and storage and has a strong flavor, while its primary oxidation product, hydrogen peroxide, is biotoxic and cytotoxic [15]. Therefore, increasing attention is being paid to the development of appropriate delivery systems for the encapsulation, protection, and release of DHA [16]. Commercial algal oil products, such as gel confectioneries, DHA drops, and microcapsules, have entered the market [17]. Gel confectioneries, which are primarily solid and require chewing, are suitable for children over 2 years of age. DHA drops, which are formulated by adding fat-soluble edible flavors and antioxidants to algal oil, often retain a marine odor, with the risk of oxidation increasing upon exposure to air after opening [18]. Furthermore, DHA microcapsules often exhibit poor organoleptic attributes and low encapsulation efficiency.

Nanoemulsions are a new type of carrier system that has been widely studied and applied in biology, medicine, pharmacology, and functional food development [19]. They are usually transparent or semi-transparent emulsions with particle sizes within the range of 50–500 nm [20]. Nanoemulsions are mainly formed by mixing water, oil, and emulsifiers in appropriate proportions to form a thermodynamically stable homogeneous dispersion system [21]. DHA oils use electrostatic modification through a coating of CS/particles, which significantly improves their chemical stability [22]. Nanoemulsions are one of the most widely used delivery systems for this purpose because they can be prepared using existing processing techniques and are easy to handle. The encapsulation of hydrophobic substances in nanoemulsions can also greatly improve their water dispersion, chemical stability, bioavailability, and bioactivity [23]. Researchers have found that the compounding effect of two emulsifiers can greatly enhance the stability of an emulsion, and the use of appropriate emulsifiers to encapsulate algal oil can mask its original flavor [24]. Therefore, nanoemulsions represent promising nanoscale delivery systems for high-DHA-content algal oil.

Consequently, the purpose of this study was to use CS as the target protein for modifying the best target polysaccharide with chitosan, maltodextrin, dextran, and GA. The nanoemulsion was finally prepared by the complex; to establish a stable algal oil emulsion carrier system in order to improve the physical and chemical stability, solubility, and bioavailability of DHA algal oil and obtain a high-quality DHA nanoemulsion; and to provide a scientific basis for the in-depth development and utilization of microalgal oil.

## 2. Materials and Methods

### 2.1. Materials

DHA algal oil was provided by Hubei Fuxing Biological Technology Co., Ltd. (Hanchuan, China). CS, glucan, maltodextrin, GA, WPI, potassium bromide, and OPA (o-phthalaldehyde) were purchased from Shanghai Yuanye Biotechnology Co., Ltd. (Shanghai, China). Chitosan was purchased from Shanghai McLean Biochemical Technology Co., Ltd. (Shanghai, China). Formaldehyde, 0.1% SDS (sodium dodecyl sulfate), a 5% sodium tetraoborate solution, β-mercaptoethanol, sodium hydroxide, n-hexane, acetone, dichloromethane, isopropanol, and 95% ethanol were purchased from Sinopharm Chemical Reagent Co., Ltd. (Shanghai, China). All of the chemicals used in this experiment were of analytical grade and were used as received unless otherwise stated.

### 2.2. Preparation of the CS–Polysaccharide Conjugated Complex

Amounts of 2.000 g of CS and 4.000 g of polysaccharides (glucan, maltodextrin, GA and chitosan) were mixed separately in a beaker, dissolved in 100 mL of ultrapure water, and placed in a magnetic stirrer (YuHua, Model DF-101S, Gongyi Yuhua Instrument Co., Ltd., ZhengZhou, China) at 1000 r/min for 2 h for premixing [25], using a 0.2 mol/L NaOH solution to adjust the pH of 8.0 of the emulsifier hydration solution. The hydrated solution was freeze-dried in a lyophilizer (JinHao, Model FD5 series, Shanghai Jinhao Scientific Instrument Co., Ltd., Shanghai, China) at −50 °C for 48 h. Water can be effectively removed via the lyophilization process, making the emulsifier more stable. Lyophilization of the emulsifier can improve its stability, extend the storage period, and improve the dissolution rate and redispersion. The lyophilized samples were removed and ground using a 100-mesh sieve. The lyophilized samples were removed and ground using a 100-mesh sieve. The sample powder was flatly spread in a Petri dish, wrapped with tinfoil, and tied with small holes, after which it was placed in a desiccator with a saturated KBr solution at the bottom, placed in a constant-temperature incubator (YiHeng, Model DHP-9902, Shanghai YiHeng Scientific Instrument Co., Ltd., Shanghai, China) to ensure that a constant humidity and temperature were maintained during the reaction, sampled and stirred every day, and reacted for a certain period of time to obtain a meladic product of CS–polysaccharides. This product was freeze-dried at −50 °C for 6 h and then placed in the refrigerator at 4 °C for storage until use [26].

#### 2.2.1. Degree of Grafting (DG) of the CS–Polysaccharide Conjugated Complex

An amount of 0.040 g of OPA was dissolved in 1 mL of formaldehyde, followed by 2.5 mL of 0.1% SDS, 25 mL of a 5% sodium tetraoborate solution, and 100 μL of β-mercaptoethanol. Finally, the OPA reagent was diluted to 50 mL with ultrapure water [27].

For the analysis, 4 mL of the OPA reagent was pipetted into a centrifuge tube, and 200 μL of the solution to be measured was injected into the centrifuge tube. This mixture was vortexed for 30 s and mixed well, and was then reacted for 2 min in a water bath at 35 °C. The absorbance was measured using a UV spectrophotometer (YouKe, Model UV-755B, Shanghai YouKe Scientific Instrument Co., Ltd., Shanghai, China) at 340 nm, and the difference △*A* between the two samples, with the addition of 200 μL of the OPA reagent and 200 μL of water as the blank sample, was taken as the net absorbance value of the free amino groups [28]. A standard curve was made with lysine, and the content of the free amino groups in the samples was calculated according to △*A*. The relative percentage of free amino groups in each sample was calculated using unmodified CS as a standard. The DG was calculated using Equation (1):

(1) $$DG(\%)=\frac{A_{0}-A_{1}}{A_{0}}\times 100\%$$

where *A*_0_ is the absorbance at 340 nm before the reaction and *A*_1_ is the absorbance at 340 nm after the reaction.

#### 2.2.2. The Determination of the Emulsion Stability Prepared by Four Emulsifiers

A sample solution with an emulsifier concentration of 1 mg/mL was prepared using a sodium phosphate buffer. A volume of 9 mL of the sample solution with 3 mL of DHA algal oil was removed and homogenized in a high-speed shear mixer (VMA, Model CV3-PlUS, VMA-Getzmann, Reichshof, Germany) at 12,000 r/min for 1 min. Then, 50 μL of the emulsion was aspirated from the bottom at 0 and 30 min, and 5 mL of an SDS solution was well mixed into the aspirated fluid [29]. The absorbance of the solution at 0 min (*A*_0_) was measured using a 0.1% SDS solution as a blank, and the absorbance of the solution was also measured after 30 min (*A*_30_). Attention was paid to the layering of lotion after 30 min, so that the lower layer of lotion must be penetrated when taking the lotion. The emulsifying activity index (EAI) indicated the ability of a protein to form an emulsion, which is closely related to the number of exposed groups and the rate of molecular migration. The emulsion stability index (ESI) could be used to evaluate the ability of emulsion droplets to maintain stability during emulsification and flocculation, and was related to the interface membrane thickness and the charge density of the interfacial surface of the droplets [30]. The EAI and ESI were calculated using Equations (2) and (3) as follows [31]:

(2) $$EAI(m^{2}g)=\frac{2\times 2.303\times A_{0}\times D}{C\times \phi \times L\times 10^{4}}$$

(3) $$ESI(\%)=\frac{A_{30}}{A_{0}}\times 100\%$$

where *A*_0_ and *A*_30_ are the absorbance values of the 100-fold diluted emulsion at 500 nm before and after 30 min of homogenization; *D* is the dilution factor; *C* is the emulsifier concentration before the emulsion preparation (g/mL); *φ* is the oil volume fraction; and *L* is the width of the optical path (1 cm).

### 2.3. Optimization of the Process for the Preparation of the Maillard Reaction Products

CS and GA were dissolved in 200 mL of ultrapure water and placed in a magnetic stirrer at 1000 r/min for 2 h for premixing [32]. The hydrated solution was frozen and placed in a lyophilizer, and the lyophilized sample was removed and ground through a 100-mesh sieve. The sample powder was spread flat in a Petri dish, placed in a desiccator with a saturated KBr solution at the bottom, and then placed in a constant-temperature incubator for the reaction. Finally, the obtained complexes were stored at 4 °C for further analysis. The effects of the CS–GA mass ratio, reaction temperature, and reaction time on the glycosylation reaction were investigated. Orthogonal tests were designed to optimize the process conditions for the preparation of the CS–GA complex, and the degree of grafting was used as the investigation index.

### 2.4. Infrared, Ultraviolet, Fluorescence, and Raman Spectroscopic Detection of Grafts

#### 2.4.1. Infrared Spectroscopy of Complexes

The sample powder was mixed with potassium bromide at a mass ratio of 1:100, ground evenly in a mortar, and pressed into sheets [33]. An infrared spectrometer (PerinElmer, Frontier, PerinElmer instruments Co., Ltd., Waltham, MA, USA) was used with a wavenumber range of 400–4000 cm^−1^, 64 scans, and a resolution of 4 cm^−1^. Potassium bromide tablets were used as blank controls, and infrared spectral acquisition was repeated at least three times for each sample under the same experimental conditions.

#### 2.4.2. Ultraviolet Spectroscopic Detection of Complexes

The sample was diluted to a solution with an emulsifier mass concentration of 1 mg/mL using distilled water, which was scanned in the range of 190–400 nm using a UV spectrophotometer. The absorbance value of the sample was determined with distilled water as a blank control [34].

#### 2.4.3. Fluorescence Spectroscopic Detection of Complexes

The fluorescence emission spectra of the solutions were obtained using fluorescence spectrophotometry (RiLi, Model F-4600, Hitachi Instruments (Suzhou) Co., Ltd., Suzhou, China) at room temperature [35]. To determine the tryptophan residue environment of the samples, the excitation wavelength was set to 295 nm, the fluorescence scanning range was 300–500 nm, the scanning speed was set to medium, and the slit width was 5 nm. The solution concentrations were maintained at 0.0001 mg/mL, and each sample solution was measured in triplicate.

#### 2.4.4. Raman Spectroscopic Detection of Complexes

The confocal microscopy Raman spectroscopy imaging system InViaTM Qontor^®^ (Renishaw, invia-qontor, Renishaw Instruments Co., Ltd., New York, NY, USA) was used, with a laser wavelength of 785 nm, an excitation power of 150 mW, and an exposure time of 10 s. A Leica microscope (10 eyepieces, 50 LWD objective lens) was also used, with a fiber-optic probe (750 nm), a spectral resolution of 1 cm^−1^, a DDCCD detector (1,024,256 pixels, 26 μm), a spectral range of 100~3200 cm^−1^, a lateral spatial resolution of 0.5 μm, a longitudinal spatial resolution of 1.5 μm, and an automatic optical platform with more than 100 mm in the XY direction. The minimum step size accuracy was 0.1 μm. Each sample was scanned more than three times, with the results being averaged by the computer, and the peak error was controlled within 3 cm^−1^ [36].

### 2.5. Thermal Stability of the Graft

#### 2.5.1. Thermogravimetric Analysis (TGA)

TGA (Maitler, Model 1100SF, METTLER TOLEDO, Kern, Grove City, OH, USA) of CS, GA, and CS–GA was performed. Argon was used as the purging gas. The samples of CS, GA, and CS–GA were weighed to obtain 5 mg portions in an alumina dish, and these portions were then heated from 25 °C to 800 °C with a temperature increase rate of 10 °C/min [37].

#### 2.5.2. Differential Scanning Calorimetry (DSC)

A DSC apparatus (Waters, Model Q2000, TA Instruments Co., Ltd., Shanghai, China) was used to analyze the thermal denaturation properties of different substances. Approximately 5 mg of each sample was weighed into an aluminum pan, the air inside the cassette was expelled, and the sample was heated in nitrogen at a rate of 10 °C/min from 25 °C to 180 °C at a flow rate of 20 mL/min of nitrogen [38]. The DSC apparatus was calibrated with indium, the empty pan was used as a reference, and the data were recorded and processed using the universal Analyze2000 software.

### 2.6. Scanning Electron Microscopy (SEM) Analysis of the Complex

CS, GA, and CS–GA powders were fixed on a sample table with conductive double-sided tape. An appropriate amount of powder was placed on the double-sided tape, the tape was placed in a scanning electron microscope (JEOL, Model JEM-2100, Japan Electronics Corporation, Beijing, China), and photos were taken. Briefly, the samples were diluted to 0.5 mg/mL with acetic acid in a buffer of 0.1 mol/L. A drop of the diluted suspension was used to coat a carbon-plated copper rack, and the rack was dried in neutral air at 25 °C. The samples were then scanned and photographed. The surface structures of the samples were observed using SEM. After freeze-drying, the samples were fixed to a copper plate and sprayed with gold. The samples were then scanned and photographed at a voltage of 15 kV.

### 2.7. Nanoemulsion Preparation

The CS, GA, CS–GA, and WPI emulsifier (0.500 g) were weighed separately, and 100 mL of deionized water was added to form a solution with a certain mass fraction, and the supernatant was centrifuged at 2500 r/min after magnetic stirring for 2 h [39]. DHA algal oil (0.500 g) was added to the obtained supernatant, and a crude emulsion was first made using high-speed shearing. Then, the crude emulsion was further processed three times using a high-pressure homogenizer (XinZhi, Model UV-2450, Ningbo Xinzhi Biotechnology Co., Ltd., NingBo, China) to obtain nanoemulsions. The nanoemulsions were treated at 50 MPa (single-stage) using a high-pressure homogenizer (flow rate of 8 L/h) equipped with a high-pressure ceramic needle-seat valve. The pre-homogenized native and denatured dispersions were further homogenized at 15 MPa with a ceramic ball-seat valve (conventional homogenization treatments, single-stage), using three channels with the same parameters.

The prepared emulsion was diluted 200 times with ultrapure water, and the particle size, polymer dispersity index (PDI), and zeta potential were determined using the nanoparticles and a zeta potential analyzer (Malvern, Model ZEN-3600, British Malvin Instruments Co., Ltd., Marvin, Britain).

### 2.8. CS–GA Nanoemulsion Preparation for Single-Factor Experiments

According to the experimental results, the main factors affecting the stability of the CS–GA emulsion were the ratio of the oil–phase, the amount of emulsifier added, and the number of homogenizations [40]. In this process, the same homogeneous pressure of 50 MPa was used for the stability test. In the univariate experiment, only one factor was changed, and the other factor was not fixed.

### 2.9. Process Optimization for the Preparation of Nanoemulsions

CS–GA (0.500 g) was weighed and dissolved in 100 mL of deionized water to form a solution, and the supernatant was centrifuged at 2500 r/min after magnetic stirring for 2 h [41]. DHA algal oil (0.500 g) was added to the obtained supernatant, and a crude emulsion was first made using high-speed shearing; then, the crude emulsion was further processed using a high-pressure homogenizer a certain number of times to obtain a nanoemulsion. We investigated the effects of the addition of an emulsifier, the oil–phase, and the number of homogenization cycles on the stability of the DHA algal oil nanoemulsions. A central composite experiment was also designed using the Design Expert 8.0.6 software to optimize the factors, with the CS–GA nanoemulsion potential as the target value. The optimal preparation process parameters for the CS–GA nanoemulsions were determined using ANOVA by evaluating the factor levels and their interactions.

### 2.10. Determination of Rheological Properties of Emulsions

A multifunctional rheometer (Waters, Model DHR-2, TA Instruments Co., Ltd., Shanghai, China) was used to determine the rheological behaviors of the CS, GA, CS–GA, and WPI nanoemulsions. For each nanoemulsion, approximately 1 mL of the newly prepared emulsion was titrated onto the iron plate. The rheometer was fitted with a 40 mm-diameter aluminum parallel plate fixture with a 1000 μm gap. Silicone oil was added to the edge of the emulsion to prevent volatilization, and a temperature control system was used to ensure that all the samples were tested at the same temperature and maintained at 25 °C with an equilibration time of 3 min before testing [26,42]. The spindle used had a disc geometry with an angle of 1° (Brookfield Engineering, Lawrence, MA, USA), and the software used was Rheo CompassDSR502 (Brookfield Engineering, Lawrence, MA, USA). For dynamic frequency scanning, the frequency range was 1~100 rad/s, the strain was 3%, and the temperature was 25 °C, so that the function curve between the energy storage modulus G′, the loss modulus G″, and the frequency was obtained according to the frequency change curve [42]. The shear rate was increased from 0.1 to 100 s^−1^ at a temperature of 25 °C, resulting in a functional curve of the relationship between the shear rate and the apparent viscosity. Each sample was scanned three times, and the computer calculated the average.

### 2.11. Determination of the Digestive Properties of DHA Nanoemulsions

#### 2.11.1. Gastric-Phase Digestion

An amount of 2.000 g of NaCl and 7 mL of hydrochloric acid were dissolved in 1 L of distilled water to form a reservoir solution. The freshly prepared emulsion (10 mL) was mixed with the reservoir solution, the pH of the mixed system was quickly adjusted to 2.5 with a 1 mol/L HCl solution, a preheating treatment was carried out for 5 min at 37 °C, 32 mg of pepsin was added, and then the solution was digested for 2 h at 37 °C in a constant-temperature water-bath oscillator (100 r/min) [43].

#### 2.11.2. Enteric-Phase Digestion

Before the simulated small-intestinal digestion, a simulated small-intestinal digest containing 24 mg/mL bile salts, 24 mg/mL lipase, and 1 mg/mL trypsin was configured with a phosphate buffer at a pH of 7.0 [44]. Immediately after the simulated gastric digestion, the pH of the sample digestion solution was adjusted to 7.0, and then an equal volume of simulated small-intestinal digestion solution was added, mixed, and adjusted to 7.0 in the system. The reaction was then placed in a constant-temperature shaker at 37 °C for 2 h.

#### 2.11.3. Determination of the Physicochemical Properties of the Nanoemulsion during In Vitro Digestion

The particle size and PDI of the nanoemulsion of the simulated stomach and simulated small-intestinal digestion at different digestion stages were determined, and the pH of the entire system was maintained at 7.0 by adding a NaOH solution in order to mimic the environment of digestion and to ensure that the enzymes function in the most appropriate environment [45].

### 2.12. Statistics and Analysis

Measurements of all the indicators were repeated three times and the results were expressed as the mean ± SD. The SPSS19 software and Excel 2019 software were used for the analyses, which included the following: one-way ANOVA, a determination of significant differences according to a Duncan’s multiple comparison analysis (lowercase letters indicated significant differences (*p* < 0.05)), a correlation analysis, and a principal component analysis. The analyses were performed using Origin2019 drawing and linear fitting of the standard curve.

## 3. Results and Discussion

### 3.1. Degree of Grafting of Multiple Polysaccharide Complexes Composed with CS

**Figure 1:**
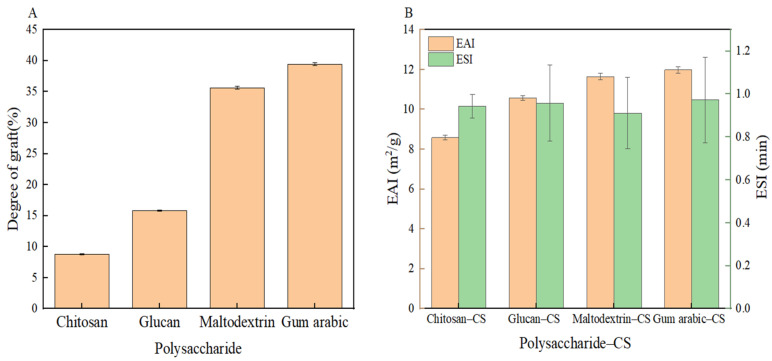
Degree of grafting of CS and different polysaccharides (**A**); emulsification properties of CS and different polysaccharides (**B**).

This study confirmed the covalent binding of chitosan, glucan, maltodextrin, and GA under tyrosinase conditions using OPA. The degree of grafting was determined by measuring the change in free amino acid content after the OPA reaction. Figure 1 shows that the degree of grafting between CS and GA was 39.45%. This study revealed that covalent crosslinking can occur between CS and GA. This cross-linking was primarily due to interactions between the carboxyl and hydroxyl groups. CS has good surface activity, stability, gelation, and hydrophilicity properties. These results suggest that the covalent linking of CS to most arabino glycan molecules had the most significant effect on improving the functional properties of CS [46].

### 3.2. Emulsifying Properties of CS–Polysaccharide Complexes

Glycosylated proteins have exceptional emulsification properties, as demonstrated by their EAI and ESI values [47]. As shown in Figure 1, the GA conjugate exhibited higher EAI and ESI values than those of the other polysaccharides. This was attributed to the macromolecular stabilizing layer formed by the covalent bond between GA and the protein around the oil droplets. The stabilizing layer prevented the emulsification, flocculation, and agglomeration of oil droplets, resulting in improved protein emulsification properties.

### 3.3. Optimization of the Preparation Process of the CS–GA Complexes

**Figure 2:**
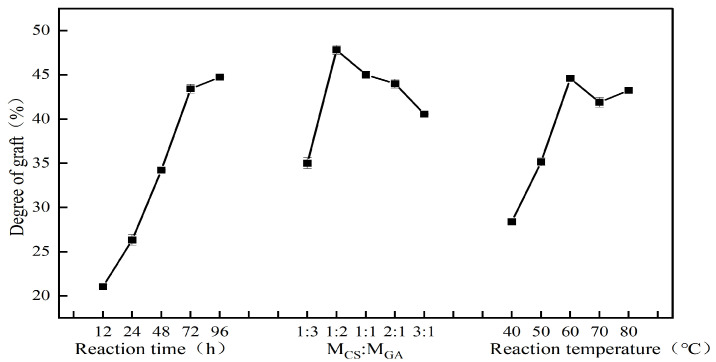
Effect of reaction factors on degree of grafting of complexes.

As shown in Figure 2, the degree of grafting with an increasing reaction time showed a gradually increasing trend, and the reaction rate was higher before 72 h, while the reaction rate decreased after 72 h. This was mainly because the amino groups of the proteins in the initial stage of the reaction were not fully exposed, the groups of GA were not in sufficient contact with the amino groups, and the exposure of the amino groups was promoted by incubation. 

Furthermore, with a decreasing GA ratio, the degree of grafting first increased and then decreased, with the maximum degree of grafting observed at a cone ratio of 1:2. When the emulsifier concentration was unchanged, with an increase in the GA concentration, the collision binding probability between the amino group inside the protein and the GA group in the system increased, and thus the reaction moved toward the grafting reaction, and the degree of grafting gradually increased. However, when the increase in the sugar concentration reached a certain extent, the spatial obstruction between the protein and the polysaccharide reduced the collision probability, which was not conducive to the progress of the reaction. Controlling the appropriate ratio can not only improve grafting, but also reduce the occurrence of side reactions, such as carbamylation. Therefore, it is appropriate to choose a CS–GA mass ratio of 1:2.

**Figure 3:**
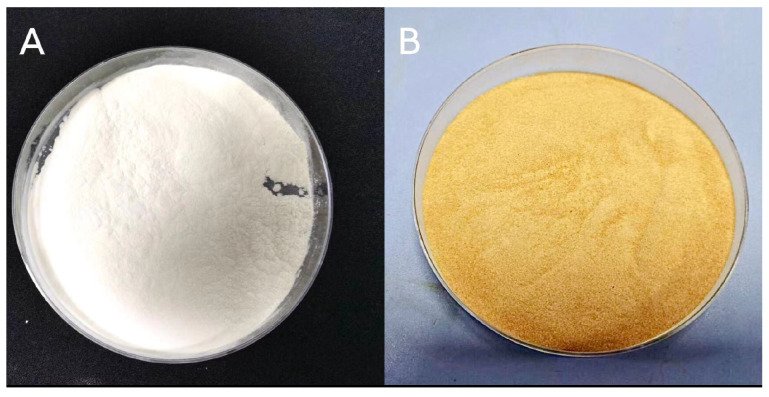
Comparison of the CS–GA complex before and after the Maillard reaction. CS–GA complex before reaction (**A**), CS–GA complex after reaction (**B**).

The degree of grafting gradually increased with an increasing reaction temperature and decreased above 60 °C (Figure 2). The degree of grafting reached its maximum value. Increasing the temperature can accelerate intermolecular movement, improve the binding of proteins and GA, and gradually increase the degree of grafting. However, with increasing temperature, the unfolding of the coiled protein revealed that the binding of more amino acids in the folded region and GA elevated the degree of the reaction. This indicated that the temperature increase favored the progress of the Maillard reaction [48]. The Maillard reaction was more fully grafted, and the prophase reaction rate was faster. Moreover, as the reaction time increased, the Maillard reaction continued to the advanced stage, which produced a brown or black color, as shown in Figure 3.

When the reaction temperature was too high, the protein exposed the disulfide bond inside the molecule and interacted with the thiol group to form aggregates, resulting in part of the amino group being buried inside. The number of amino acids that could bind to GA decreased, resulting in a reduction in grafting. Scholars at home and abroad prepared the complexes using dry or wet methods under different reaction conditions and investigated their functional properties. Sultan et al. used rice protein hydrolysate and dextran to prepare a complex that showed significant improvements in solubility and the emulsification of rice protein [49].

On the basis of one-way experiments, a three-factor, four-level L_16_ (4^3^) orthogonal experiment was conducted to optimize the process conditions for the preparation of CS–GA complexes. The degree of grafting was investigated using various CS–GA ratios (1:3, 1:2, 1:1, 2:1, and 3:1), reaction temperatures (40 °C, 50 °C, 60 °C, 70 °C, and 80 °C), and reaction times (12 h, 24 h, 48 h, 72 h, and 96 h). The degree of grafting was used as the index. The orthogonal table is shown in Table 1.

The results showed that the factor with the greatest effect on the degree of grafting of the CS–GA complexes was the reaction time, followed by the reaction temperature and the CS–GA ratio. Consequently, the optimal conditions for obtaining the maximum degree of grafting were as follows: a 96 h reaction time, a 1:2 CS–GA ratio, and a 60 °C reaction temperature. Three parallel validation tests were conducted under the optimal test conditions, resulting in an average degree of grafting of 44.91% for CS–GA.

### 3.4. UV Spectral Analysis of the Complex

**Figure 4:**
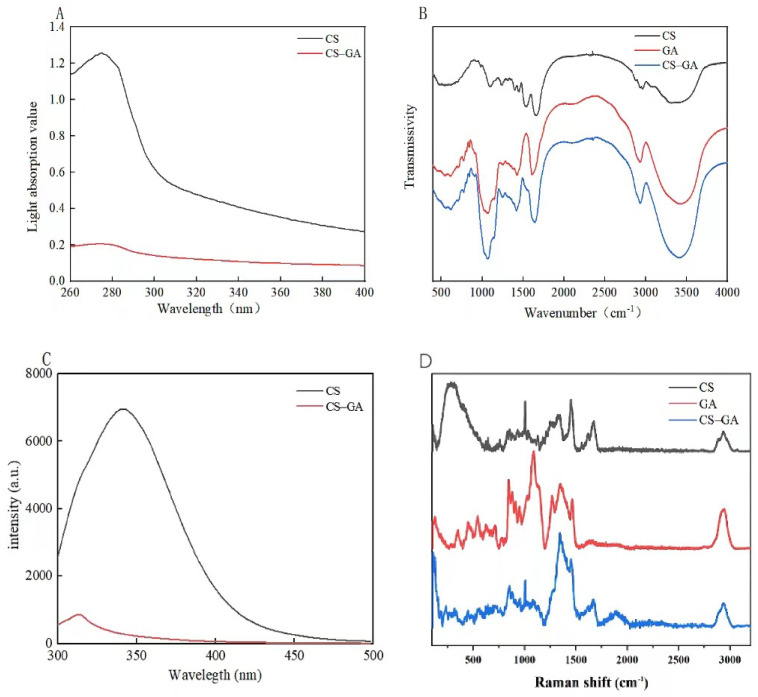
UV (**A**), IR (**B**), fluorescence (**C**), and Raman (**D**) spectroscopy analyses of CS, GA, and CS–GA.

The UV absorption spectrum is a method used to study Maillard products, and the junction can be judged according to the different UV absorptions and whether the structure is changed [50]. The UV absorption spectra of the grafts with different degrees of reaction are shown in Figure 4. The figure shows a distinct characteristic peak with a maximum absorption wavelength of approximately 280 nm. The absorption peak at 250–300 nm is related to the aromatic amino acids (such as tryptophan, tyrosine, and phenylalanine) in CS and the chromogroups (such as pyran, furan, and pyrrole) produced during the Maillard reaction. When CS formed a complex with GA, the intensity of the characteristic UV absorption peak of the CS–GA complex solution around 280 nm was greatly reduced at the same concentration of solution, which indirectly indicated the generation of a complex between CS and GA.

### 3.5. An IR Spectroscopy Analysis of the Complex

Fourier transform infrared spectroscopy is important for detecting the structure of proteins and dipeptides and the composition of food components [51]. As shown in Figure 4, in the infrared spectrum of proteins, the absorption peaks in the range of 3300–3600 cm^−1^ originated from N-H stretching vibrations and O-H stretching vibrations, and the weak band around 2900 cm^−1^ was due to C-H stretching and bending vibrations. Characteristic absorption peaks within 1600–1700 cm^−1^, 1530–1550 cm^−1^, and 1260–1300 cm^−1^ correspond to amide I, amide, and amide bands, respectively, and are related to N-H bending vibrations, C-N stretching vibrations, and C=O stretching vibrations. The absorption peaks within 1040–1150 cm^−1^ originated from O-H deformation vibrations and C-O stretching vibrations.

At the 3300–3600 cm^−1^ band, the absorption peak of the CS–GA complex is shifted to the right, and the characteristic absorption peak of the amide I band (1600–1700 cm^−1^) in the protein indicated changes in the chemical group and secondary structure of the protein. CS was assigned to amide I at 1656.41 cm^−1^. The characteristic absorption peak at 1539.11 cm^−1^ was attributed to the amide band. The CS–GA composite’s amide I band was shifted to 1639.41 cm^−1^, and the characteristic absorption peak of the amide band disappeared. At 3300–3600 cm^−1^, the conjugated hydroxoyl stretch vibration was more intense than in CS. At 1040 cm^−1^, the presence of a hydroxyl bend in the conjugate Qu vibration was indicated, which may have been related to the coupling of GA to CS. The above analysis confirmed the Maillard reaction between CS and GA.

### 3.6. Raman Spectral Analysis of the Complex

Raman spectroscopy is often used to study complexes of proteins and polysaccharides, where the intensity changes and displacement of the bands reflect the changes in the corresponding groups or chemical bonds. When a group or chemical bond is broken or damaged, the molecular vibration energy level changes, leading to a decrease in the intensity and a shift in the position of the peaks. As shown in Figure 4, the intensity of amide I of the CS–GA complex was lower than that of casein, indicating that the CS–GA complex had an unfolded shape compared to casein. The Fermi resonance of tyrosine caused a change in the characteristic peaks near 850 cm^−1^ and 830 cm^−1^ in the Raman spectra of proteins, and the specific behavior was shown to change with the microenvironment of the protein’s side chain. The apparent double peaks at 830 cm^−1^ and 850 cm^−1^ were due to tyrosine absorption vibrations, and the increased intensity of the bands at 1460 cm^−1^ for the complexes compared to CS and GA may have been due to the action of polysaccharides, which resulted in the amino acid residues being buried inside the molecule. The binding of CS and GA was further confirmed.

### 3.7. Endogenous Fluorescence Spectral Analysis of the Complex

As a technique for measuring binding affinity, fluorescence quenching is an effective method for detecting protein conformational changes as well as complex formation [52]. Therefore, to further validate the results of UV absorption spectroscopy, fluorescence spectroscopy was used to investigate the changes in the fluorescence emission spectra of the tryptophan (Trp) residues after the binding of CS to GA. As shown in Figure 4, CS showed a maximum fluorescence emission at 341 nm under 295 nm excitation. However, the CS–GA complex exhibited a lower intensity. These results suggest that polysaccharides exert a shielding effect when bound to proteins. From the results of this study, it can be speculated that certain fluorescent intermediate compounds may polymerize to form brown pigments with different chemical structures, resulting in the lower fluorescence of the intermediate compounds. In addition, the maximum fluorescence emission peak position of CS–GA was shifted to 313 nm, which may have been due to a change in the hydrophobicity around the tryptophan residues in the CS molecule and peptide as a result of binding with GA.

### 3.8. Thermal Stability Analysis of the Complex

**Figure 5:**
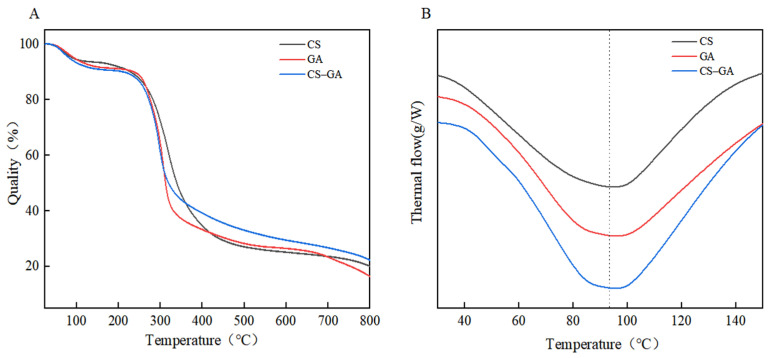
CS, GA, and CS–GA thermogravimetric analyses (**A**); differential scanning calorimetry (**B**: Vertical lines indicate thermal absorption peaks).

Protein samples are generally not heat-stable, and food emulsions as final products need to undergo a high-temperature pasteurization process. Therefore, the thermal stability of the prepared emulsions affects their stability and product properties. The thermal stability of CS, GA, and CS–GA under nitrogen was evaluated using a thermogravimetric analyzer, as shown in Figure 5. The TGA curves of CS, GA, and CS–GA showed a decreasing trend below 100 °C, which may have been due to the evaporation of water molecules on the surface. The temperatures at which the thermal decomposition rates of the three reached the maximum were basically the same, in the range of 300–310 °C. From the weight loss temperature, it can be seen that the stability was in the order of CS–GA > CS > GA, while the maximum thermal decomposition rate was in the order of CS–GA > CS > GA. Therefore, the thermal stability of the CS–GA complex was improved compared with CS and GA. This may have been due to the fact that the binding of the sugar moiety to the protein hinders the thermal aggregation and weakens the thermal absorption of the peptide chain, thus improving its thermal stability.

DSC is typically used to characterize complexes by comparing the thermal behavior of individual components and complexes [53]. The DSC results showed that many thermal transformations occurred when the samples were heated. Under the preset conditions of the instrument, more significant thermal absorption peaks were observed for all three compounds, and the thermal denaturation temperatures (TDT) and initial denaturation temperatures (IDT) of the CS–GA complex were significantly higher than those of CS and GA. The enthalpy change of CS–GA was 231.4 J/g, as shown in Table 2. It was hypothesized that additional non-covalent interactions generated during the formation of the CS–GA complexes induce a significant change in enthalpy and that the increase in the denaturation temperature of the CS–GA complexes may be related to the incorporation of GA leading to the α-helix structure reduction.

### 3.9. Appearance Representation of the Complexes

**Figure 6:**
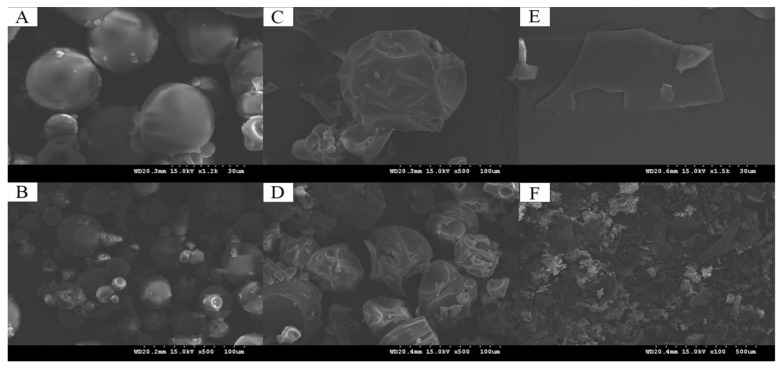
SEM of microstructural changes in CS (**A**: magnification of 1200×; **B**: magnification of 500×), GA (**C**: magnification of 500×; **D**: magnification of 500×), and CS–GA (**E**: magnification of 1500×; **F**: magnification of 100×) powders after freeze-drying.

Freeze-drying is an effective method for prolonging the storage time of samples. SEM is an important tool for studying the structure of nanomaterials and is often used for the structural and morphological characterization of composites to observe their morphological changes before and after synthesis [54]. Microstructural changes in the CS, GA, and CS–GA powders after freeze-drying were observed using SEM (Figure 6). We observed significant differences in the microstructures of the three samples. CS exhibited a spherical structure with smooth surfaces of different sizes, a high porosity, and different pore sizes, while GA had an irregular spherical structure. In contrast, CS–GA exhibited a more obvious lamellar structure with closer cross-linking between protein molecules, indicating that the binding of CS and GA was more stable and able to induce the protein molecules by altering the intermolecular interactions of CS to induce the binding of protein molecules with GA, resulting in the formation of complexes with a regular morphology.

### 3.10. Single-Factor Condition and the Response Surface Analysis of the Effect of the Nanoemulsions

**Figure 7:**
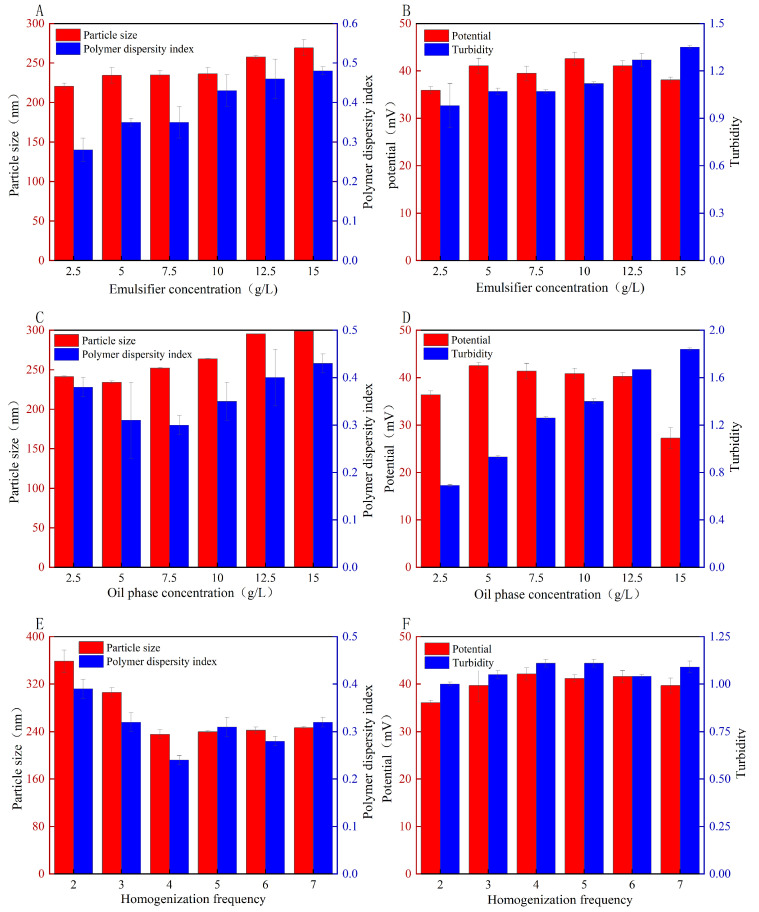
Univariate condition analysis of effect of emulsion. Emulsifier concentration (**A**,**B**), oil–phase concentration (**C**,**D**), and homogenization frequency (**E**,**F**).

CS–GA was used as an emulsifier to prepare nanoemulsions with buried DHA algal oil. The molecular structure of the nanoemulsions included both hydrophilic and oleophilic groups, which can significantly change the interface state of the nanoemulsion system and prevent the separation of the two phases. Therefore, the selection of an appropriate emulsifier can effectively improve the stability of emulsifying systems. The amount of added emulsifier affected the strength of the interfacial membrane formed on the surface of the dispersed phase droplets. As shown in Figure 7, when the amount of DHA algal oil was fixed, the particle size, dispersion coefficient, and turbidity of the nanoemulsions increased. However, when the amount of emulsifier increased from 5 g/L to 10 g/L, the emulsions particles were smaller, the distribution of the particle sizes was narrower, and the potentials had a tendency to increase and then decrease. When the amount of emulsifier was low, the interfacial film could not form on the surface of the droplets; thus, nanoemulsions were formed with small particle sizes. When the amount of emulsifier increased from 5 g/L to 10 g/L, the emulsion particles were smaller, the distribution of the particle sizes was narrower, the potentials had a tendency to increase and then decrease, and the absolute values of the potentials were all below 40 mV, compared with those at lower and higher emulsifier concentrations.

As shown in Figure 7, the particle size and turbidity of the nanoemulsions increased with the oil content. When the amount of emulsifier was fixed and the amount of DHA algal oil was small, some emulsifiers could not form nanoemulsions with the DHA oil. When the nanoemulsions were fixed and the DHA oil was in excess, some DHA algal oil could not be emulsified by the emulsifier, the emulsified oil droplet volume increased, and the contact area with water tended to decrease because of collision aggregation among the oil droplet particles. The dispersion coefficient and potential of the nanoemulsions initially decreased and then increased. When the amount of DHA was 5 g/L, the nanoemulsion had the largest absolute value and the smallest particle size. Therefore, three oil–phase concentrations of 2.5 g/L, 5 g/L, and 7.5 g/L were selected for the optimization experiment.

The fixed homogenization pressure was 300 MPa, and the number of homogenizations was varied to obtain the particle size, PDI, zeta potential, and turbidity of the CS–GA nanoemulsions, as shown in Figure 7. With an increase in the number of homogenizations, the nanoemulsion size and dispersion coefficient exhibited a gradually smooth trend, and the potential of the nanoemulsions was reduced after the increasing trend. When the number of homogenizations was five, the potential had the largest absolute value, and the homogenization number continued to increase the potential gradually. This may have been because, with an increase in the homogenization times, mechanical energy was converted into heat energy, and the nanoemulsion absorption phase transition occurred so that the nanoemulsion stability changed, the system nanoemulsion structure was destroyed, and the stability of the newly formed nanoparticles was reduced. Therefore, homogenization frequencies of four, five, and six were selected for the optimization experiments.

**Figure 8:**
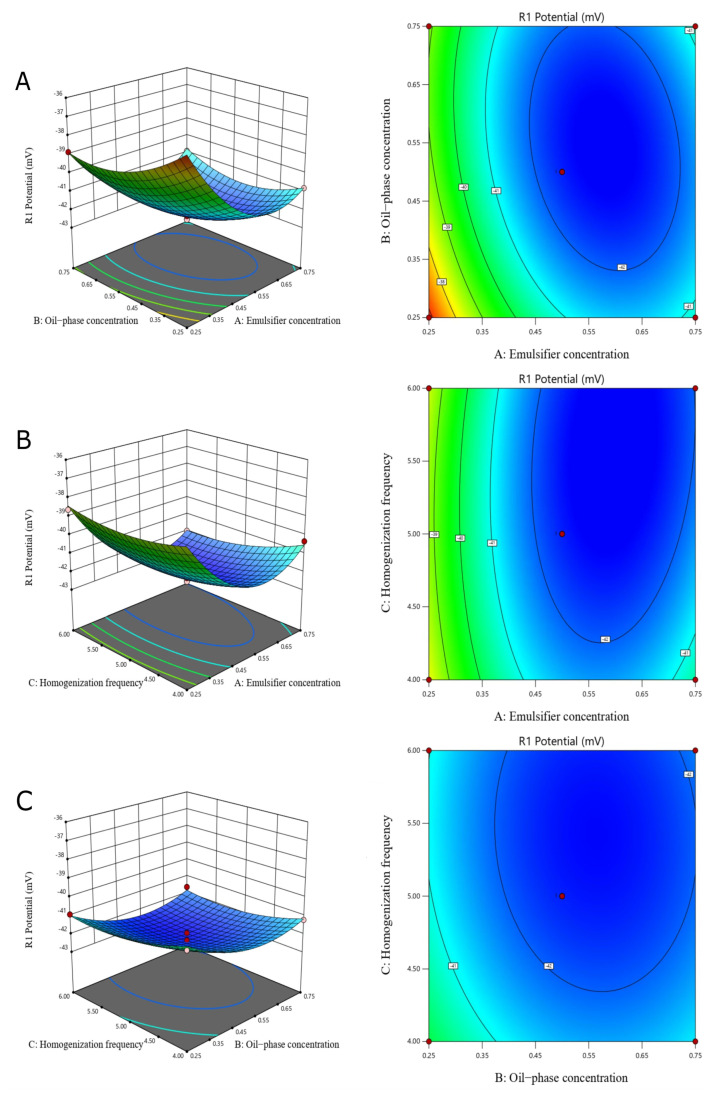
Response surface analysis of emulsions (The red dots indicate Design points, the different colors indicate the range of potentials, from blue to red indicates the potentials from small to large). Response surface and contours of interaction between water and oil concentrations (**A**); response surface and contours of interaction between emulsifier concentration and homogeneity frequency (**B**); and response surfaces and contours for interaction between oil–phase concentration and homogenization frequency (**C**).

Response surface optimization was carried out to investigate the effects of the three factors—the emulsifier-phase concentration, the oil–phase concentration, and the number of homogenizations—on the stability of the nanoemulsions, using the potential as an indicator. Figure 8 shows the response surface curve and contour for each interaction factor.

The experimental results showed that the regression equations could be used to analyze and predict the optimal process conditions for the preparation of CS–GA DHA algal oil nanoemulsions—that is, an emulsifier concentration of 5 g/L, an oil–phase concentration of 5 g/L, and five homogenizations. The response surface experimental design is shown in Table 3 and the regression equation ANOVA analysis is shown in Table 4. After three validation experiments, the emulsion potential was −42.35 mV, with a relative error of 1.1% with respect to the theoretical prediction, which indicated that the regression model is reliable.

### 3.11. Analysis of the Rheological Properties of the Four Emulsion Types

For nanoemulsion systems applied to food, understanding their rheological characteristics is helpful for improving their nutrition and quality. Therefore, it is of great interest to investigate the stability and structure of emulsions through rheological testing. G′ represents the amount of energy that can be recovered by the system during the shear deformation cycle, while G″ represents the amount of energy consumed, representing the solid and liquid properties of the system, respectively. When G′ > G″, the system exhibits elastic deformation, i.e., solid, and when the opposite is true, it exhibits viscous deformation, i.e., liquid.

**Figure 9:**
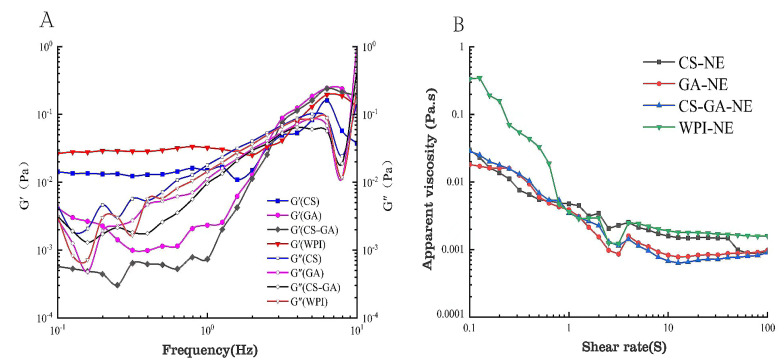
Rheological properties of CS, GA, CS–GA, and WPI emulsions. Variation of the modulus of emulsions prepared with different emulsifiers with oscillation frequency (**A**) and the apparent viscosity of emulsions prepared with different emulsifiers (**B**).

Kim [55] found that after 21 d of storage, emulsions with an inner layer of WPI and an outer layer of fish gelatin (FG) had better oxidative stability than emulsions with the reverse order of adsorption. By comparing the emulsifier synthesized based on the Maillard reaction with the commonly used WPI, the performance and merits of the new synthetic emulsifier can be evaluated. As can be seen from Figure 9A, the energy storage modulus and the loss modulus increased with the frequency, indicating that the modulus has a strong frequency dependence. G′ > G″ for CS, and the WPI emulsion showed more reversible deformation than irreversible deformation, while having elastic behavior and certain gel properties, and at higher frequencies, G″ > G′ showed higher liquid properties. GA-NE and CS–GA-NE were the opposite, with G′ > G″ at higher frequencies and better fluid properties at lower frequencies. Figure 9B shows the apparent viscosity of the four emulsions, which shows shear thinning behavior—that is, the apparent viscosity of the emulsion decreased with increasing shear rate. This indicated that the emulsion was a typical non-Newtonian pseudoplastic fluid, and that the high shear rate disrupted the interfacial force between the emulsion droplets. As the shear rate increased from approximately 0.1 to 10 s, the apparent viscosity decreased sharply, and the decrease in the shear viscosity was consistent with changes in the droplet or polymer structure in the emulsion [56]. A higher shear force may have caused the apparent viscosity because of the following: (1) the flocs in the emulsion may have been destroyed; (2) the polysaccharide network in the emulsion may have been destroyed [57]; or (3) the droplets or polymers may have aligned with the shear field rather than being randomly distributed. Compared with CS, GA, and CS–GA, WPI had a better apparent viscosity at each shear rate, and the overall apparent viscosity of CS-NE, GA-NE, and CS–GA-NE was relatively consistent at the same concentration.

### 3.12. Digestion Characteristics of Nanoemulsions

**Figure 10:**
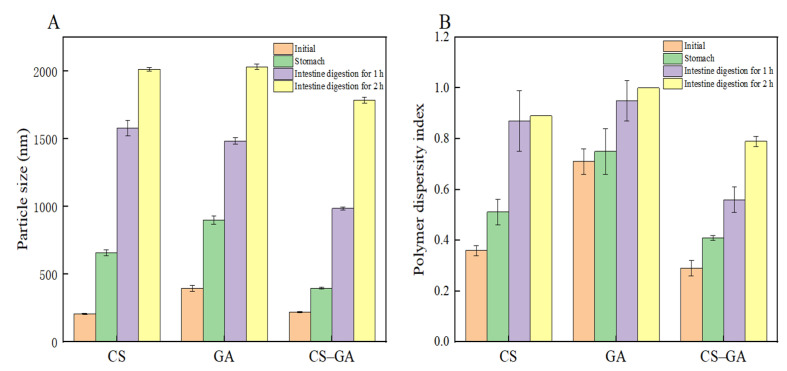
Mean particle size (**A**) and polymer dispersity index (**B**) of DHA algal oil emulsion before and after in vitro digestion.

In the gastric digestion stage, the results of which can be seen in Figure 10, four average emulsion sizes increased significantly. The main components in gastric juice were simulated, including inorganic salts and pepsin. The solution was highly acidic, with a pH of only 2.5, although compared with traditional nano-emulsions, it was smaller due to the influence of polar conditions. The strong acid in the solution enabled aggregation in the emulsion because the electrostatic repulsion force between the emulsion droplets became smaller, and the pH and ion interactions increased the emulsion aggregation and particle size. In addition, there was a certain impact caused by the effect of the strong acid; the simulated gastric juice contained a large number of hydrogen ions, and these hydrogen ions and the nanoparticles underwent group neutralization, thus resulting in a loss of negative charge. This made the nanoparticles more inclined to show a certain hydrophilic behavior, thus greatly reducing the hydrophobic ability of the oil surface of the droplets. As a result, the emulsion became unstable. The potential of the emulsion was reduced, which indicated that the emulsion’s structure was damaged by the strong acidic environment.

In the intestinal digestion phase, the size of the particles in the emulsion further increased. Even when the solution was restored to a neutral pH, the emulsion underwent further decomposition, the potential of the emulsion continued to decrease, and the dispersion coefficient also further increased in the intestinal digestion phase. This is because the emulsion included bile salts, lipids, and a number of other negatively charged substances, which have strong surface activity. Therefore, they can adsorb to the surface of the emulsion, and because the negative charge of these substances is smaller than that of the protein, the charge gradually becomes lower than that of the gastric digestion phase.

## 4. Conclusions

Firstly, GA was selected as the best modified polysaccharide from the four polysaccharides based on the degree of grafting index, and the optimal process for the best complex was obtained after optimization with a reaction time of 96 h, a CS−GA mass ratio of 1:2, and a reaction temperature of 60 °C. Under Fourier infrared spectroscopy, the characteristic absorption peak of CS−GA attributed to the amide I band at 1656.41 cm^−1^ was shifted to 1639.41 cm^−1^, and the characteristic absorption peak attributed to the amide II band at 1539.11 cm^−1^ disappeared. In the Raman spectrum, the intensity of the bands of the complexes increased at 1460 cm^−1^ compared to GA. In the UV spectrum, the intensity of the characteristic UV absorption peak around 280 nm of the complex was greatly reduced compared with that of CS. In the fluorescence spectrum, the maximum emission wavelength of tryptophan of the complex was shifted from 341 nm to 313 nm compared with that of CS, and the fluorescence intensity was significantly reduced, which proved that the introduction of GA caused the change in the secondary structure of CS. The thermal decomposition temperature of the complex was high as detected by TGA, and the enthalpy change value of the complex was 231.4 J/g under DSC, which was much larger than that of CS and GA, and it had good thermal stability. By optimizing the preparation process of emulsion, the optimal preparation process of the emulsion was obtained as follows: an emulsifier-phase concentration of 5 g/L, an oil–phase concentration of 5 g/L, and five homogenizations. From the rheological experiments, the emulsifier has certain colloidal properties at higher frequencies (G′ > G″) and better fluid properties at lower frequencies. It can maintain a relatively stable structure in the in vitro simulated digestion experiment. As a nanoscale transmission system, nanoemulsions represent a promising DHA algal oil transmission system that can load a high content of DHA algal oil. The selection of appropriate emulsifiers for burying algal oil can allow the original flavor of the algal oil to be largely hidden and can improve its physical and chemical stability. In this study, the coupling of polysaccharides to CS involved only grafting, emulsification activity, and emulsification stability. The properties of a stable CS emulsion should be further studied to broaden the knowledge on this emulsifier.

## Figures and Tables

**Table 1:** The orthogonal experimental design and the results (the CS–GA ratio, reaction temperature, and reaction time were considered the grafting index).

Experiment Number	A—Time (h)	B—CS:GA Ratio (g)	C—Temperature (°C)	Degree of Grafting/%
1	1	1	1	19.11
2	1	2	2	28.90
3	1	3	3	26.01
4	1	4	4	25.09
5	2	1	2	31.21
6	2	2	1	25.55
7	2	3	4	29.11
8	2	4	3	33.34
9	3	1	3	34.22
10	3	2	4	44.00
11	3	3	1	32.11
12	3	4	2	43.24
13	4	1	4	35.19
14	4	2	3	44.74
15	4	3	2	44.89
16	4	4	1	39.29
k1	24.777	29.932	29.015	
k2	29.803	35.797	37.060	
k3	38.392	33.030	34.578	
k4	41.028	35.240	33.347	

**Table 2:** Thermal denaturation parameters of CS, GA, and CS–GA.

Samples	IDT (°C)	TDT (°C)	ΔH (J/g)
CS	36.09	94.92	165.50
GA	46.45	93.70	181.00
CS–GA	47.64	95.47	231.40

**Table 3:** Response surface experimental design and experimental results (the potential index investigated the emulsifier concentration, oil–phase concentration, and homogenization frequency).

Experiment Number	A—Emulsifier Concentration (g/L)	B—Oil–phase Concentration (g/L)	C—Homogenization Frequency	Potential (mV)
1	2.5	2.5	5	−36.68
2	7.5	2.5	5	−40.80
3	2.5	7.5	5	−38.85
4	7.5	7.5	5	−40.92
5	2.5	5	4	−38.18
6	7.5	5	4	−40.32
7	2.5	5	6	−38.62
8	7.5	5	6	−42.02
9	5	2.5	4	−40.20
10	5	7.5	4	−41.22
11	5	2.5	6	−40.92
12	5	7.5	6	−41.65
13	5	5	5	−42.51
14	5	5	5	−41.95
15	5	5	5	−42.36
16	5	5	5	−42.54
17	5	5	5	−42.56

**Table 4:** Regression equation variance analysis (the F-value is equal to the ratio of the mean squared error between groups and the mean squared error within groups, which reflects the magnitude of the effect of random error).

Source of Variance	Sum of Squares	Degree of Freedom	Mean Squares	F	*p*
Model	47.63	9	5.29	78.51	<0.0001 **
A—Emulsifier concentration	17.2	1	17.2	255.13	<0.0001 **
B—Oil–phase concentration	2.04	1	2.04	30.26	0.0009 **
C—Homogenization frequency	1.35	1	1.35	20.07	0.0029 **
AB	1.05	1	1.05	15.58	0.0055 **
AC	0.4	1	0.4	5.89	0.0457 *
BC	0.021	1	0.021	0.31	0.5939
A^2^	19.32	1	19.32	286.57	<0.0001 **
B^2^	3.64	1	3.64	53.96	0.0002 **
C^2^	0.88	1	0.88	13.04	0.0086 **
Residual term	0.47	7	0.067		
Unplanned item	0.21	3	0.071	1.09	0.4508
Pure error	0.26	4	0.065		
Sum	48.11	16			

Note: * indicates a significance level of *p* < 0.05; ** indicates a high significance level of *p* < 0.01.

## Data Availability

The original contributions presented in the study are included in the article; further inquiries can be directed to the corresponding author.
